# Impact of physical indicators on ocular development in preschool children

**DOI:** 10.3389/fmed.2024.1483852

**Published:** 2024-11-19

**Authors:** Xiangxiang Liu, Jing Fu, Lei Li, Peipei Liu, Yunyun Sun, Huijian Li, Yuanbin Li, Bidan Zhu, Shana Wang, Xi Qin

**Affiliations:** ^1^Beijing Tongren Eye Center, Beijing Tongren Hospital, Beijing Ophthalmology & Visual Sciences Key Laboratory, Capital Medical University, Beijing, China; ^2^Tongzhou Maternal and Child Health Hospital of Beijing, Beijing, China

**Keywords:** height, weight, body mass index, visual acuity, refraction

## Abstract

**Objective:**

Understanding the impact of early childhood physical growth on visual development is crucial, as this period marks a critical phase for foundational physical and ocular maturation. The aim of the current study was to investigate the associations between the anthropometric indicators of height, weight, and body mass index (BMI), as well as visual acuity, refraction, and ocular biometrics, in Chinese preschool children.

**Methods:**

This cross-sectional study consisted of 1,477 Chinese 3- to 6-year-old preschool children from nine kindergartens in Tongzhou District, Beijing. Demographic data, height and weight were measured according to a standard protocol, and BMI was calculated. Refractive error was measured via autorefraction in eyes under cycloplegia. Axial length (AL), anterior chamber depth (ACD), lens thickness, and corneal curvature were measured via an IOL Master. The axial length–corneal radius (AL–CR ratio) was defined as the AL divided by the mean corneal radius of curvature. Multivariate linear regression models were used to explore the cross-sectional associations between physical indicators (height, weight and BMI) and visual acuity and ocular developmental parameters in boys and girls.

**Results:**

Compared with the children in the fourth quartile for height for a given age and sex, the visual acuity in the fourth quartile was 0.08 less, the refraction was 0.11 D more negative (1.22 D versus 1.33 D), the axial length was 0.62 mm longer, the anterior chamber depth was 0.18 mm deeper, the lens thickness was 0.13 mm thinner, the corneal radius of curvature was 0.1 mm less, and the AL-CR ratio was higher after adjustments were made for age and weight. The association between BMI and visual acuity was statistically significant in girls but not in boys. Older and more obese children had better visual acuity (*p* < 0.001) after adjustments were made for age.

**Conclusion:**

Height and higher BMI remained independently related to VA condition, AL and ACD elongation, and corneal flattening in preschool children after controlling for various covariates. These results provide critical insights into pediatric ocular health and emphasize the importance of early detection and intervention in both physical and ocular health in early childhood development.

## Introduction

Child development is a multifaceted process in which vision and eye health play critical roles (1, 2). Vision is not only important for a child’s learning abilities and social skills but also closely linked to their overall physical and mental health (3). Preschool children are in a critical phase of physiological development, during which their visual system rapidly develops. This process is characterized by the formation and strengthening of neural pathways that are essential for basic visual functions, such as depth perception, color vision, and eye coordination (4, 5). Early detection and treatment of visual impairments are crucial during this stage, as they are highly responsive to correction and adaptation (6).

Among eye diseases, myopia is the most pressing global issue and may affect children’s visual acuity (1). Recent epidemiological data show a significant increase in its prevalence, particularly during and after the COVID-19 pandemic (7–9). Studies indicate that the prevalence of myopia among school-aged children has nearly doubled, from approximately 13% between 2015 and 2019 to 25% in 2021 (10). The increase in myopia is attributed to lifestyle changes during the pandemic, such as increased near-work and screen time and reduced outdoor activities (11, 12). These findings highlight the impact of environmental factors on myopia development in children. The pandemic’s restrictions exacerbated these issues (8, 13). A timely understanding of children’s eye development is crucial for subsequent public eye health management. However, few studies have focused on the visual development of preschool children, especially after the pandemic.

Additionally, recent studies have shown that the COVID-19 lockdown has resulted in notable changes in children’s physical growth patterns due to decreased activity and altered dietary habits (14–16). A systematic review and meta-analysis revealed a significant increase in body weight and body mass index (BMI) among school-aged children and adolescents during the lockdown (17). Previous studies have shown a complex relationship between physical growth indicators, such as height and weight, and eye development (18). As the critical period for physical and ocular development occurs in early childhood, it is invaluable to study the effects of dynamic changes in physical development on the process of visual development during this period (19). Fewer studies have been conducted in preschool-aged children. Therefore, the current study investigated the differences between ocular biometrics and anthropometric determinants, including height, weight, and body mass index, in preschool-aged Chinese children. This research not only provides insight into the critical period of visual system development in preschool children but also addresses the demanding issue of the potential impact of altered growth patterns on children’s eye health. This study also highlights the importance of early detection and intervention to alleviate visual impairments in this age group, providing a scientific basis for further public health measures.

## Materials and methods

### Study population

A continuous prospective study, which included children from nine kindergartens in the Beijing Tongzhou district, has been conducted annually since 2021. The screening examinations are performed from December–January each year. Ethical approval for this project was granted by the Beijing Tongren Hospital Ethical Committee in accordance with the Declaration of Helsinki. Participation in the study necessitated obtaining written consent from parents and guardians and verbal consent from children, without any financial incentives. The purpose of this study is to present the first cross-sectional data focusing on physical indicators (weight, height, BMI), visual information and ocular biometric data and the associations between these variables and the age of the children determined at the time of screening. Following the Strengthening the Reporting of Observational Studies in Epidemiology (STROBE) guidelines, this study provides transparent and comprehensive reporting of its observational methodology. Participants with ocular abnormalities, previous ocular surgery or trauma, or systemic diseases that could affect vision were excluded.

### Procedures

Each child underwent basic systemic examinations and a series of eye examinations. Body weight and height were measured, and BMI was calculated as the ratio of body weight in kilograms divided by the square of body height in meters.

Uncorrected visual acuity (UCVA) was measured as described previously (20). In brief, UCVA was measured monocularly (right followed by left eye) via Lea Symbols 3-m Set charts (250,300, Goodlite, Elgin, IL, USA) illuminated under standard room lighting. The participants read the numbers on the right of each line sequentially, starting from the top, until they made an error. If a mistake was made, they were instructed to resume reading from two lines above the point of error. Reading continued until three or more errors were made. The score for the detailed logMAR VA was determined by calculating the errors on the final line and preceding lines, which were determined on a letter-by-letter basis.

Refractive errors were measured before and after cycloplegia using a calibrated autorefractor (KR-800, Topcon), with each assessment performed three times to obtain an average reading. To ensure accurate measurement of refractive errors, 1% cyclopentolate was employed. Cycloplegic refraction was conducted using a standardized protocol for all patients (21, 22). The refractive error readings were obtained 15 min after ensuring full cycloplegia. The spherical equivalent refraction (SER) was defined as the sum of the spherical power and half of the cylindrical power.

Ocular alignment assessments were performed via the Hirschberg light reflex test, along with alternate and cover-uncover tests. The fixation targets were placed at 6 meters and at a near range of 33 centimeters for accurate measurements.

Axial length, anterior chamber depth, lens thickness, corneal curvature radius, and central corneal thickness were measured via biometry (Lenstar LS900; Haag-Streit Koeniz, Switzerland). The axial length–corneal radius (AL–CR ratio) was defined as the AL divided by the mean corneal radius of curvature. Other eye examinations included slit lamp examination (SL-3G; Topcon, Tokyo, Japan) and intraocular pressure (CT-800; Topcon, Tokyo, Japan) to exclude ocular abnormalities.

### Data analysis

The axial length, anterior chamber depth, lens thickness, central corneal thickness, corneal curvature radius, and SER were normally distributed. There was a high degree of consistency between the data from both eyes, with Pearson correlation coefficients of 0.95 for refractive error and 0.97 for axial length, resulting in the presentation of results from the left eye only. The height, weight and BMI distributions were categorized into quartiles and further analyzed by sex and specific age groups, namely, 3-, 4-, 5- and 6-year-old boys and girls, as shown in Table 1.

**Table 1:** Quartiles and means of height, weight, and BMI by age and sex.

	Boys				Girls			
	3 Years	4 Years	5 Years	6 Years	3 Years	4 Years	5 Years	6 Years
Height (cm)								
First quartile	≤98.9	≤104.0	≤111.4	≤115.8	≤98.8	≤103.4	≤110.0	≤114.0
Second quartile	99.0–101.7	104.1–107.6	111.5–114.4	115.9–119.0	98.9–102.0	103.5–107.0	110.1–113.5	114.1–117.5
Third quartile	101.8–105.0	107.7–111.0	114.5–118.3	119.1–122.0	102.1–105.0	107.1–111.0	113.6–117.0	117.6–120.3
Forth quartile	>105.0	>111.0	>118.3	>122.0	>105.0	>111.0	>117.0	>120.3
Mean ± SD	101.9 ± 4.5	107.7 ± 5.1	114.8 ± 5.2	118.8 ± 5.5	101.7 ± 3.9	107.1 ± 5.1	113.6 ± 5.1	117.1 ± 4.6
Median	101.7	107.6	114.4	119.0	102.0	107.0	113.5	117.5
(Range)	92.4–113.6	96.0–124.7	100.5–129.0	105.0–133.5	93.6–110.4	94.6–121.0	100.0–131.9	105.8–126.6
Weight (kg)								
First quartile	≤15.3	≤16.8	≤19.0	≤20.2	≤15.0	≤16.6	≤18.3	≤19.5
Second quartile	15.4–16.5	16.9–18.4	19.1–20.8	20.3–22.4	15.1–16.0	16.7–18.0	18.4–19.9	19.6–21.5
Third quartile	16.6–18.3	18.5–20.0	20.9–23.1	22.5–24.8	16.1–17.3	18.1–19.3	20.0–21.7	21.6–23.9
Forth quartile	>18.3	>20.0	>23.1	>24.8	>17.3	>19.3	>21.7	>23.9
Mean ± SD	17.0 ± 2.4	19.1 ± 4.0	21.4 ± 4.0	23.2 ± 4.1	16.3 ± 2.1	18.3 ± 2.5	20.4 ± 3.3	22.2 ± 3.8
Median	16.5	18.4	20.8	22.4	16.0	18.0	19.9	21.5
(Range)	12.5–24.5	13.4–38.0	19.0–20.8	16.5–35.5	12.7–24.5	13.0–31.0	14.0–35.5	16.5–33.0
BMI (kg/m^2^)								
First quartile	≤15.3	≤15.0	≤14.9	≤14.9	≤14.8	≤14.9	≤14.6	≤15.0
Second quartile	15.4–16.2	15.1–15.9	15.0–15.7	15.0–16.0	14.9–15.6	15.0–15.7	14.7–15.5	15.1–15.7
Third quartile	16.3–17.1	16.0–16.7	15.8–17.0	16.1–17.1	15.7–16.4	15.8–16.6	15.6–16.5	15.8–16.8
Forth quartile	>17.1	>16.7	>17.0	>17.1	>16.4	>16.6	>16.5	>16.8
Mean ± SD	16.3 ± 1.6	16.4 ± 2.9	16.2 ± 2.4	16.3 ± 1.9	15.7 ± 1.5	15.8 ± 1.4	15.7 ± 1.7	16.2 ± 2.2
Median	16.2	15.9	15.7	16.0	15.6	15.7	15.5	15.7
(Range)	12.7–22.8	11.3–33.8	15.7–17.0	13.2–22.1	12.9–21.6	12.2–23.0	12.4–23.7	12.2–23.6

Consecutive integers were assigned to each quartile to conduct linear trend analyses, with dependent variables regressed against these integers to evaluate the significance of the slope of the score variable. Outcome variables, such as biometry readings, corneal curvature, refractive error, and the AL–CR ratio, were examined through multivariate linear regression, and the study population’s characteristics were adjusted to assess correlations between the children’s growth measurements and clinical parameters in boys and girls. All the statistical analyses were performed via SPSS software (SPSS for Mac, v.23.0; IBM-SPSS, Chicago, Illinois, USA), and two-sided *p* < 0.05 was considered statistically significant.

## Results

A total of 1,515 children aged 3–6 years were included in the study. Data from a total of 38 children were excluded either because their ocular findings were considered to affect visual acuity (e.g., corneal scarring, previous surgery) or because data from both eyes were missing. Therefore, data from 1,477 children (1,477 left eyes) are presented in this analysis.

The distribution of physical measurements in a group of children aged 3 to 6 years, categorized by age and sex, is shown in Table 1. There were 203 3-year-olds, 497 4-year-olds, 645 5-year-olds and 132 6-year-olds; 52.5% were boys, and 47.5% were girls. The age- and sex-specific quartiles of height, weight, and BMI are also shown in Table 1. As expected, boys were taller than girls, and older children were taller and heavier. Specifically, the mean height and weight of boys were 101.9 ± 4.5 cm and 17.0 ± 2.4 kg in 3-year-olds, respectively; 107.7 ± 5.1 cm and 19.1 ± 4.0 kg in 4-year-olds, respectively; 114.8 ± 5.2 cm and 21.4 ± 4.0 kg in 5-year-olds, respectively; and 118.8 ± 5.5 cm and 23.2 ± 4.1 kg in 6-year-olds, respectively. The mean height and weight of girls were 101.7 ± 3.9 cm and 16.3 ± 2.1 kg in 3-year-olds, respectively; 107.1 ± 5.1 cm and 18.3 ± 2.5 kg in 4-year-olds, respectively; 113.6 ± 5.1 cm and 20.4 ± 3.3 kg in 5-year-olds, respectively; and 117.1 ± 4.6 cm and 22.2 ± 3.8 kg in 6-year-olds, respectively.

**Figure 1:**
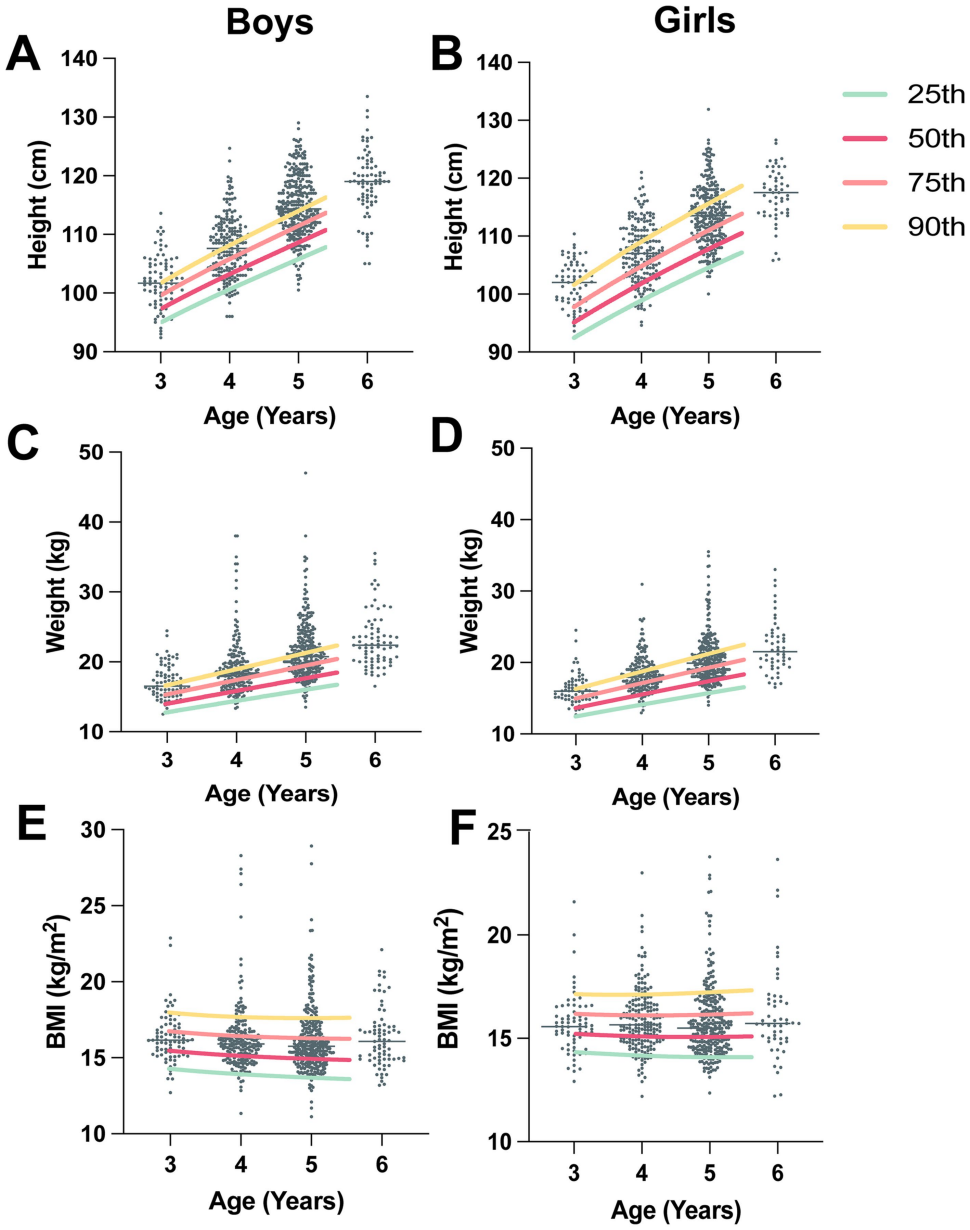
Distributions of children’s height, weight and BMI categorized by age and sex. Each graph is overlaid with percentile lines indicating the 25th, 50th, 75th, and 90th percentiles, illustrating the WHO recommended child growth standards (3 to 5 years). **(A)** The distribution of boys’ height and the WHO-recommended height-for-boy (3–5-year percentile) curve. **(B)** Distribution of girls’ height and the WHO-recommended height-for-girl (3–5-year percentile) curve. **(C)** The distribution of boys’ weight and the WHO-recommended weight-for-boy (3–5-year percentile) curve. **(D)** The distribution of girls’ weight and the WHO recommended weight-for-girl (3–5-year percentile) curve. **(E)** The distribution of boys’ BMI and the WHO-recommended BMI-for-boy (3–5-year percentile) curve. **(F)** Distribution of girls’ BMI and the WHO-recommended BMI-for-girl (3–5-year percentile) curve.

The distribution of the children’s height, weight and BMI stratified by age and the WHO recommended growth standards for children are all presented in Figure 1. As shown in Figure 1, the mean height of the children was approximately 90% of the WHO-recommended height for children of the same age, with no sex difference. The mean weight of 3-year-old children was 90% of the WHO recommended height, whereas it was approximately 75% in 6-year-old children. The mean BMI of the children was between 50 and 75% of the WHO recommended standard for all age groups.

In general, taller and heavier children had eyes with longer axial lengths, deeper anterior chambers, thinner lenses, flatter corneas, and higher AL–CR ratios compared with shorter children (Table 2). The children in the fourth quartile had eyes with a VA that was 0.08 mm less, axial lengths that were 0.62 mm longer, anterior chambers that were 0.18 mm deeper, lens thicknesses that were 0.13 thinner, flatter corneas (7.83 vs. 7.73 mm), and AL–CR ratios that were 0.04 greater (all *p* < 0.001). Eyes in children with weights in the fourth quartile had a lower VA, axial length 0.55 mm longer, anterior chambers 0.14 mm deeper, lens thickness 0.08 thinner, flatter corneas (7.82 vs. 7.70 mm), and AL–CR ratios 0.03 greater than those in children in the first quartile (*p* < 0.01). In addition, children with higher BMIs tended to have better VAs (0.22 vs. 0.20, *p* = 0.023), longer axial lengths (22.35 mm vs. 22.17 mm, *p* = 0.031), and greater CCTs (by 4.07 μm; *p* = 0.037).

**Table 2:** Visual acuity, refraction and ocular biometry measurements by quartiles of height, weight and BMI.

	*n*	Visual acuity	SE (D)	Axial length (mm)	Anterior chamber depth (mm)	Lens thickness (mm)	CCT (um)	Corneal curvature (mm)	AL-CR ratio
Height (cm)									
First quartile	381	0.26 ± 0.11	1.33 ± 0.89	21.94 ± 0.62	2.65 ± 0.27	3.83 ± 0.22	542.75 ± 38.64	7.73 ± 0.25	2.84 ± 0.06
Second quartile	361	0.22 ± 0.11	1.25 ± 0.84	22.17 ± 0.62	2.72 ± 0.26	3.78 ± 0.25	544.05 ± 34.08	7.74 ± 0.24	2.86 ± 0.07
Third quartile	378	0.20 ± 0.09	1.31 ± 0.83	22.36 ± 0.67	2.77 ± 0.23	3.74 ± 0.22	541.42 ± 34.03	7.79 ± 0.27	2.87 ± 0.06
Forth quartile	357	0.18 ± 0.13	1.22 ± 0.83	22.56 ± 0.70	2.83 ± 0.25	3.70 ± 0.21	538.98 ± 33.09	7.83 ± 0.24	2.88 ± 0.07
*p* (trend)		**<0.001**	0.169	**<0.001**	**<0.001**	**<0.001**	0.116	**<0.001**	**<0.001**
Reg coefficient		−0.004	0.003	0.037	0.009	−0.007	−0.192	0.006	0.002
*p* (reg)		**<0.001**	**0.035**	**<0.001**	**<0.001**	**<0.001**	0.173	**<0.001**	**<0.001**
Weight (kg)									
First quartile	371	0.25 ± 0.11	1.28 ± 0.88	21.94 ± 0.62	2.67 ± 0.26	3.81 ± 0.22	542.20 ± 37.82	7.70 ± 0.25	2.84 ± 0.06
Second quartile	373	0.21 ± 0.10	1.36 ± 0.80	22.18 ± 0.63	2.72 ± 0.25	3.77 ± 0.24	541.60 ± 34.92	7.76 ± 0.24	2.85 ± 0.07
Third quartile	366	0.21 ± 0.11	1.24 ± 0.86	22.39 ± 0.68	2.78 ± 0.25	3.74 ± 0.23	541.87 ± 32.55	7.81 ± 0.26	2.87 ± 0.06
Forth quartile	367	0.20 ± 0.11	1.24 ± 0.85	22.49 ± 0.70	2.81 ± 0.28	3.73 ± 0.22	541.66 ± 34.99	7.82 ± 0.25	2.87 ± 0.08
*p* (trend)		**<0.001**	0.318	**<0.001**	**<0.001**	**<0.001**	0.878	**<0.001**	**<0.001**
Reg coefficient		−0.004	−0.006	0.050	0.013	−0.009	0.106	0.009	0.003
*p* (reg)		**<0.001**	0.286	**<0.001**	**<0.001**	**<0.001**	0.676	**<0.001**	**<0.001**
BMI (kg/m^2^)									
First quartile	368	0.20 ± 0.11	1.24 ± 0.75	22.17 ± 0.71	2.74 ± 0.23	3.74 ± 0.21	539.91 ± 32.58	7.74 ± 0.26	2.86 ± 0.07
Second quartile	371	0.21 ± 0.11	1.28 ± 0.85	22.21 ± 0.66	2.73 ± 0.28	3.77 ± 0.24	538.52 ± 35.70	7.76 ± 0.26	2.86 ± 0.07
Third quartile	369	0.22 ± 0.11	1.28 ± 0.92	22.25 ± 0.73	2.76 ± 0.25	3.76 ± 0.23	546.19 ± 37.39	7.78 ± 0.26	2.86 ± 0.07
Forth quartile	369	0.22 ± 0.13	1.31 ± 0.86	22.35 ± 0.73	2.75 ± 0.28	3.76 ± 0.24	543.98 ± 34.78	7.80 ± 0.25	2.86 ± 0.08
*p* (trend)		**0.003**	0.319	**0.006**	0.501	0.484	**0.036**	**0.002**	0.938
Reg coefficient		0.003	0.005	0.020	0.006	−0.003	0.960	0.006	0.001
*p* (reg)		**0.023**	0.653	**0.031**	0.073	0.361	**0.037**	0.090	0.622

Mean and regression coefficients for height adjusted for age, sex, and weight; mean and regression coefficients for weight adjusted for age, sex, and height; mean and regression coefficients for BMI adjusted for age and sex. Reg, regression; SE, spherical equivalent. Values in bold indicate statistical significance.

Table 3 illustrates the associations between children’s growth measurements and ocular biometric parameters in boys and girls separately. In general, children’s ocular parameters increase as their bodies develop. According to these multiple linear regression models, taller and heavier children had eyes with significantly better VAs, longer axial lengths, deeper anterior chamber depths, flatter corneas and higher AL–CR ratios (all *p* < 0.05). Moreover, taller boys but not taller girls whose eyes were refractive tended toward emmetropia (*p* = 0.015). In addition, heavier boys have thinner lenses than heavier girls (*p* < 0.001), with boys being 1 kg heavier, and their lens thickness tending to be 0.01 mm thinner. Girls with higher BMIs had eyes with significantly better VA (*p* = 0.016).

**Table 3:** Linear regression models of visual acuity, refraction and biometry measurements by height, weight, and BMI for boys and girls separately.

	Visual acuity		Refractive error (D)		SE (D)		Axial length (mm)	
	Regression coefficient (95% CI)	*p*	Regression coefficient (95% CI)	*p*	Regression coefficient (95% CI)	*p*	Regression coefficient (95% CI)	*p*
Height (cm)								
Boys	−0.004 (−0.005, −0.003)	**<0.001**	−0.001 (−0.009, 0.008)	0.869	−0.011 (−0.020, −0.002)	**0.015**	0.037 (0.030, 0.043)	**<0.001**
Girls	−0.005 (−0.007, −0.004)	**<0.001**	−0.002 (−0.009, 0.006)	0.667	−0.001 (−0.011, 0.010)	0.893	0.032 (0.025, 0.039)	**<0.001**
Weight (kg)								
Boys	−0.003 (−0.005, −0.001)	**0.007**	0.001 (−0.014, 0.016)	0.913	−0.013 (−0.028, 0.002)	0.089	0.041 (0.029, 0.053)	**<0.001**
Girls	−0.005 (−0.008, −0.002)	**<0.001**	0.001 (−0.014, 0.017)	0.855	0.010 (−0.010, 0.030)	0.325	0.046 (0.032, 0.061)	**<0.001**
BMI (kg/m^2^)								
Boys	0.003 (−0.001, 0.007)	0.111	0.004 (−0.022, 0.029)	0.775	−0.03 (−0.029, 0.023)	0.825	0.006 (−0.015, 0.026)	0.580
Girls	0.007 (0.001, 0.012)	**0.016**	0.010 (−0.021, 0.041)	0.532	0.036 (−0.005, 0.077)	0.082	0.006 (−0.025, 0.036)	0.709
	Anterior chamber depth (mm)		Lens thickness (mm)		Corneal curvature (mm)		AL-CR ratio	
	Regression coefficient (95% CI)	*p*	Regression coefficient (95% CI)	*p*	Regression coefficient (95% CI)	*p*	Regression coefficient (95% CI)	*p*
Height (cm)								
Boys	0.009 (0.006, 0.012)	**<0.001**	−0.009 (−0.011, −0.006)	**<0.001**	0.007 (0.004, 0.009)	**<0.001**	0.002 (0.002, 0.003)	**<0.001**
Girls	0.008 (0.005, 0.011)	**<0.001**	−0.004, (−0.007, −0.001)	**0.005**	0.005 (0.003, 0.008)	**<0.001**	0.002 (0.001, 0.003)	**<0.001**
Weight (kg)								
Boys	0.011 (0.007, 0.016)	**<0.001**	−0.010 (−0.015, −0.006)	**<0.001**	0.006 (0.001, 0.010)	**0.015**	0.003 (0.002, 0.004)	**<0.001**
Girls	0.010, (0.005, 0.016)	**<0.001**	−0.005, (−0.011, 0.001)	0.081	0.010 (0.004, 0.016)	**0.001**	0.002 (0.001, 0.004)	**0.003**
BMI (kg/m^2^)								
Boys	0.007 (−0.001, 0.015)	0.093	−0.003 (−0.010, 0.005)	0.472	−0.002 (−0.010, 0.006)	0.671	0.001 (−0.001, 0.004)	0.215
Girls	−0.002 (−0.014, 0.009)	0.687	0.006 (−0.005, 0.017)	0.302	0.007 (−0.005, 0.018)	0.271	−0.003 (−0.006, 0.000)	0.093

The regression coefficients are derived from a single model of multiple linear regression. In this model, each ocular biometric aspect is the dependent variable, and height, weight, or BMI is the independent variable. Adjustments in this model vary depending on the independent variable. When height was the independent variable, adjustments were made for age, sex, and height. For weight, adjustments include age, sex, and weight. For BMI, the model adjusts for age and sex. SE, spherical equivalent. Values in bold indicate statistical significance.

## Discussion

To the best of our knowledge, this is the first study to investigate the associations among visual acuity, ocular biometry measures, and child growth in preschool children. This study demonstrated that taller and heavier children tended to have certain ocular biometric parameters associated with better visual acuity, longer axial lengths, deeper anterior chamber depths, thinner lenses, flatter corneas, and higher AL–CR ratios. Furthermore, sex differences were observed in the various anthropometric measures of ocular biometry. Taller boys tended toward emmetropia, whereas heavier boys had thinner lenses. Additionally, girls with higher BMIs exhibited better visual acuity. These findings highlight the relationship between measures of physical growth and ocular development in preschool children and emphasize the importance of considering both physical and ocular health during early childhood development.

Recent studies have shown that COVID-19 lockdown restrictions have had a significant effect on the physical growth of children, resulting in changes in their height and weight (14, 23, 24). Research in Chinese preschool children revealed an overall increase in height percentiles from 2019 to 2022 (25), although the growth rate was lower during the lockdown years (26). This study also revealed a decrease in the overweight rate postlockdown, possibly due to dietary changes (27). Furthermore, a study of Chinese children aged 3–6 years reported increasing height percentiles from 2019 to 2022, with weight percentiles peaking during major lockdowns (25). The median BMI remained stable, except for a decrease in 2021, reflecting changes in weight percentiles (28). Our findings are consistent with those of previous studies. Taken together, these findings suggest that the COVID-19 lockdown had a significant effect on children’s physical development, with variations depending on age, sex, and location.

We presented the distributions of children’s height, weight, and BMI in relation to the growth standards recommended by the WHO (29). The mean height for both boys and girls approached approximately 90% of the WHO-recommended height for their respective ages, indicating a generally favorable trend in height development. The mean weight of 3-year-olds reached 90% of the WHO recommendation. However, it decreased to approximately 75% among the 6-year-olds, indicating a potential deviation from the expected weight standards with increasing age. Additionally, the mean BMI of children across all age groups fell within the range of 50 to 75% of the WHO recommended standards. These findings indicate that although height development is close to WHO standards, there may be variations in weight and BMI as children grow older.

**Figure 2:**
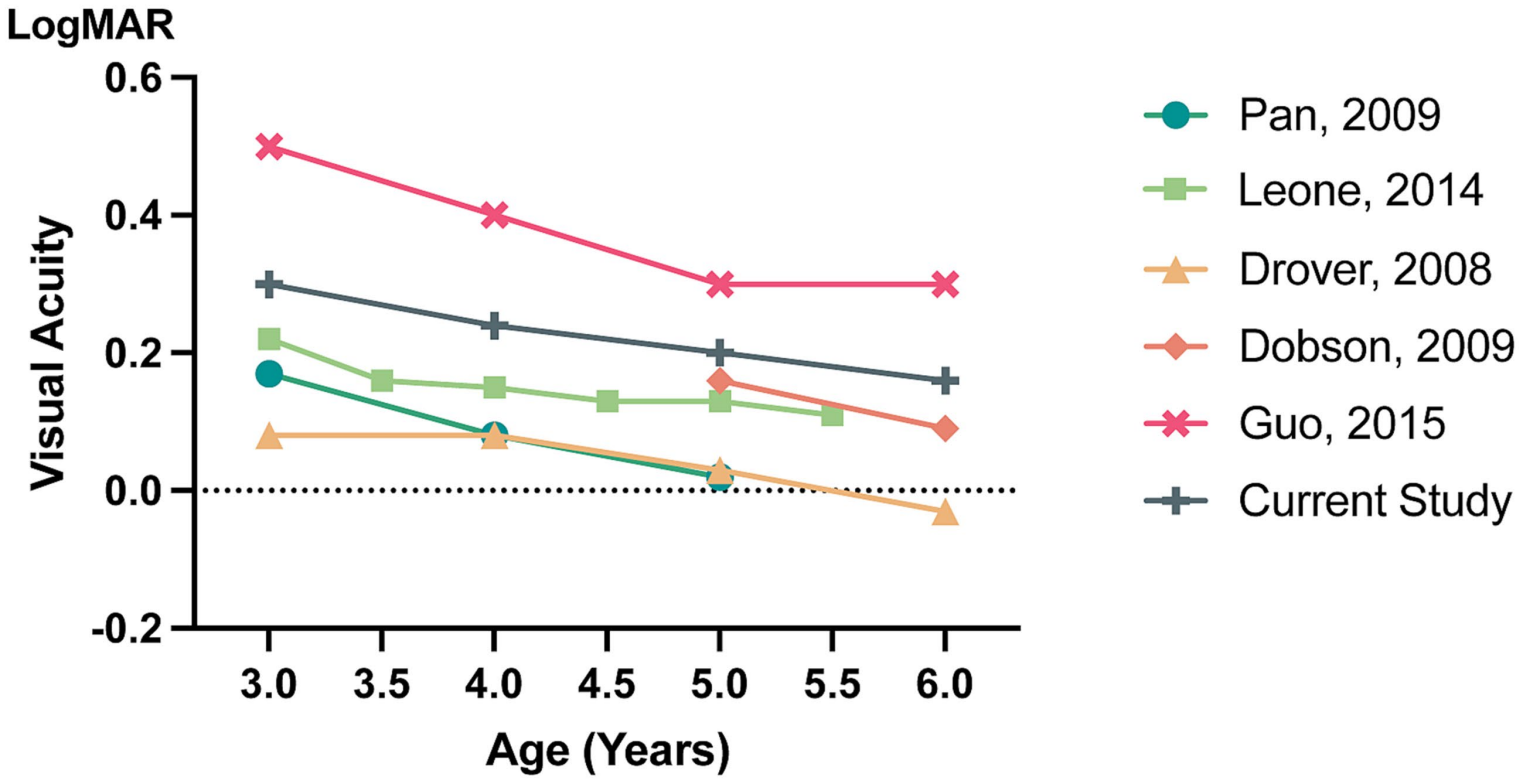
Age-specific distributions of the 95th percentile VA from epidemiological studies.

Figure 2 summarizes the age-specific distributions of the 95th percentile VA from epidemic studies (30–34). Across all studies, visual acuity improved with increasing age. However, the current study revealed a less significant improvement in visual acuity with age compared with other studies, suggesting that children in this particular cohort may have a slower rate of visual development. Possible reasons for this may include different genetic backgrounds, different VA measurements and the influence of COVID-19 pandemic restrictions.

Recent studies have confirmed an increase in myopia prevalence among school-age children during the COVID-19 epidemic (35–37). These findings indicate that the pandemic posed significant challenges to vision and eye health, particularly among younger children, who may be more susceptible to environmental changes than older children (13, 15). In addition to reduced outdoor activities and increased screen time, the discontinuation of spectacles or the treatment of amblyopia and the lack of routine eye examinations may have also affected children’s visual development (38). These findings highlight the importance and urgency of eye examinations for young children after the COVID-19 lockdown.

Both our study and previous research have revealed significant correlations between ocular biometrics and various systemic biological factors (39–41). In children, age-related changes in axial length and vitreous chamber depth affect the spherical equivalent and lens thickness. Demographic factors were found to influence ocular measurements in Ethiopian adults (42). Specifically, there was a positive correlation between axial length and anterior chamber depth and a negative correlation with lens thickness (43). Similarly, a study on Chinese schoolchildren highlighted the relationships between anthropometric indicators and ocular characteristics, emphasizing the impact of physical measurements on eye health (44). These findings demonstrate the intricate relationships between ocular biometrics and various demographic and physical characteristics, offering valuable insights for ophthalmological research and clinical practice.

Several studies have synthesized the complex relationships among height, weight and children’s visual development (44–46). Research has demonstrated a correlation between height and ocular biometrics, such as axial length and anterior chamber depth, which affect the eye’s refractive power and visual acuity. Additionally, eyes in obese children are more likely to have hyperopic refractions (44). Nutritional factors may play dual roles in promoting physical growth and ocular health (47). This is supported by several pediatric studies (48). Additionally, genetic influences may impact both child growth and ocular characteristics through common genetic pathways (49). Environmental factors, such as outdoor activities, have also been linked to physical growth and a lower risk of myopia (50). These findings, which are drawn from a range of studies, highlight the complex interplay between a child’s physical growth and ocular health. This emphasizes the need for a comprehensive approach to understanding and managing pediatric development.

Several associations between various anthropometric indices and specific biometric parameters differ between boys and girls. Taller boys, but not girls, tend toward emmetropia, suggesting possible gender differences in the visual development mechanism in young children. However, a previous study revealed that heavier boys had eyes with refractions that tended toward hyperopia and shorter vitreous chambers. The variation in results could be attributed to the difference in the age of the subjects between the two studies (44). Our study focused on preschoolers, whereas the other study examined school-aged children. This variation also implies a difference in the growth and developmental characteristics of children at different ages.

The primary limitation of this study lies in its limited generalizability due to potential selection bias. The participant cohort consisted exclusively of preschool children from nine kindergartens in Tongzhou District, Beijing, which may not adequately represent the broader population of Chinese preschool children. Furthermore, the nonrandomized selection of participants may introduce geographic, socioeconomic, or cultural factors specific to this cohort that could influence the observed associations between physical growth and ocular development. Additionally, the cross-sectional design of the study precludes the establishment of causal relationships, as it captures data at a single point in time rather than across a developmental continuum. These limitations should be taken into consideration when interpreting the study’s findings and their potential applicability to other populations.

## Conclusion

In conclusion, the interplay among child development, eye health, and vision is intricate and significant. The current study revealed that height and weight are associated with eyes with longer eyeballs, deeper anterior chambers, and corneal flattening in preschool-aged Chinese children. Eyes in taller preschool-aged boys tended to exhibit emmetropia, and heavier children had eyes with thinner lenses. These relationships varied with sex. The associations in preschool-aged children differ from recent findings in school-aged children and adults. These findings suggest that a focused analysis of anthropometric dimensions, sex, and eye growth may offer a new approach to understanding the mechanisms underlying visual development in this age group.

## Data Availability

The raw data supporting the conclusions of this article will be made available by the authors, without undue reservation.
